# Publication trends in corneal transplantation: a bibliometric analysis

**DOI:** 10.1186/s12886-016-0379-x

**Published:** 2016-11-08

**Authors:** Evre Pekel, Gökhan Pekel

**Affiliations:** 1 Denizli State Hospital, Eye Clinic, Denizli, Turkey; 2 Ophthalmology Department, Pamukkale University, Denizli, 20070 Turkey

**Keywords:** Corneal transplantation, Keratoplasty, Keratoprosthesis, Bibliometric analysis

## Abstract

**Background:**

Our aim was to demonstrate the publication trends of corneal transplantation in the last decade.

**Methods:**

All of the keratoplasty research articles, letters, case reports, reviews and meeting abstracts published between January 2006 and December 2015 indexed on the Thomson Reuters Web of Knowledge were evaluated. A bibliometric filter was used to capture keratoplasty related publications by using the key words ‘keratoplasty’, ‘corneal transplantation’ or ‘keratoprosthesis’ in the ‘title’ selection mode.

**Results:**

A total of 2726 publications were evaluated in the present study. Documents related to penetrating keratoplasty only have been decreased, whereas the documents related to endothelial keratoplasty were increased in the last decade. The total keratoplasty publication counts had been increased from the year 2006 to 2015. The average citation count per keratoplasty documents was 9.34.

**Conclusions:**

There is a growing interest to the lamellar keratoplasty techniques especially the endothelial keratoplasty in the last decade.

## Background

Corneal transplantation or keratoplasty is one of the most common transplantations performed in humans [1]. Keratoplasty is the main treatment modality for visual rehabilitation in eyes with deteriorated corneal clarity. In the last decade, corneal transplantation techniques have been rapidly evolved. There is an increase in selective replacement of the diseased corneal layer, instead of full-thickness corneal transplantation [2]. Lamellar keratoplasty techniques offer better visual outcomes and less tissue rejection [3, 4].

Bibliometric analysis is a method to quantitatively analyze academic literature in which citation reports and content analysis are mainly used [5–7]. Patterns of academic publications, geographical and institutional distribution of articles in a specific field can be evaluated by bibliometrics [5–7]. Amount of bibliometric studies related to ophthalmology practice is not much in the literature. In the present study, the bibliometric analysis of keratoplasty was performed. The mapping of the literature related to keratoplasty was done in several aspects including citations, origins of the papers and content of the publications.

Following the changes of trends in clinical practice, publication trends may also change in a similar fashion. Since newer techniques possess more unknown aspects, it is likely to find more publications related to them when compared to older techniques (i.e. penetrating keratoplasty). In the present study we aimed to demonstrate publication trends of keratoplasty in the last decade. To our knowledge, this is the first study about bibliometric analysis of corneal transplantation in the literature.

## Methods

We analyzed all keratoplasty research articles, letters, case reports, reviews and meeting abstracts published between January 2006 and December 2015 indexed on the Thomson Reuters Web of Knowledge. We created a bibliometric filter to capture keratoplasty related publications from the Thomson Reuters database by using the key words ‘keratoplasty’, ‘corneal transplantation’ or ‘keratoprosthesis’ in the ‘title’ selection mode. All the publications which met the above criteria were examined and categorized according to the type of corneal transplantation technique (i.e. penetrating, lamellar, endothelial, keratoprosthesis, mixed, animal and miscellaneous reports). The search was performed on January 2016. Inclusion/exclusion of the documents was evaluated by the two authors (EP, GP). In order to avoid discrepancies, the authors evaluated the documents together at the same time and recorded the publication category by consensus. There were no restrictions in the aspect of language. The present manuscript does not report on or involve the use of any animal or human data or tissue, and ethics approval and consent for publication are not applicable to this submission.

Penetrating keratoplasty category represented all the aspects of the technique (laser assisted, traditional mechanical trephination, etc.). Lamellar corneal transplantation mainly included anterior lamellar keratoplasty. Endothelial keratoplasty included descemet stripping automated endothelial keratoplasty (DSAEK) and descemet membrane endothelial keratoplasty (DMEK). If experiments were performed on animals, these types of documents were categorized under ‘animal studies’. All types of artificial cornea researches were categorized under ‘keratoprosthesis’ section. Publications which included more than one corneal transplantation techniques were categorized under ‘mixed studies’ division. Reports related to immunology, pharmacotherapy and eye-banking issues were categorized under ‘miscellaneous’ division.

The present bibliometric study also included quantitative data about document types, authors of the publications, journals that published the keratoplasty documents, languages, origins of the publications and citation reports. The publications related to corneal amniotic membrane transplantation, corneal limbal epithelium stem cell transplantation, corneal keratocyte transplantation, conductive keratoplasty, thermal keratoplasty and microwave keratoplasty were excluded. Unpublished documents were excluded wherever published documents on the same study were available.

In this bibliometric study, we focused mostly on a graphical representation of the data rather than statistical calculations. Microsoft Excel 2007 was used to draw graphics. In addition, we used the Statistical Package for the Social Sciences (SPSS) 17.0 software for Windows (SPSS Inc., Chicago, IL, USA) to analyze yearly quantitative distributions of different keratoplasty techniques. The Pearson correlation test was performed for this purpose. *P* values lower than 0.05 were considered to be statistically significant.

## Results

A total of 2726 publications were evaluated in the present study. Table 1 demonstrates the distribution of the number of publications according to the corneal transplantation techniques. The number of keratoplasty publications was doubled in the year 2015 when compared to the year 2006. The proportion of the documents related to penetrating keratoplasty only decreased from 49 to 15 %, whereas the proportion of the documents related to endothelial keratoplasty increased from 15 to 35 % in the last decade. The yearly quantitative distributions of document categories were highly correlated in the last 5 years (*r* > 0.90, *p* < 0.05). Table 2 shows the quantitative distribution and proportions of the publications according to the document types and study design by year. From the year 2006 to 2015, the proportion of original articles & case reports decreased from 85 to 63.5 %. The proportion of meeting abstracts markedly increased in the year 2015.

**Table 1:** Quantitative distribution and proportions of the publications according to the corneal transplantation techniques and procedures

Year	Penetr.	Lamel.	Endoth.	Keratopr.	Mixed	Animal	Miscell.	Total
2006	78 (49 %)	20 (13 %)	23 (15 %)	11 (7 %)	11 (7 %)	6 (4 %)	8 (5 %)	157
2007	89 (44 %)	36 (18 %)	38 (19 %)	10 (5 %)	9 (4.5 %)	9 (4.5 %)	10 (5 %)	201
2008	80 (34 %)	31 (13 %)	82 (%35)	15 (6 %)	9 (4 %)	8 (3 %)	12 (5 %)	237
2009	78 (31 %)	33 (13 %)	98 (38 %)	13 (5 %)	17 (7 %)	3 (1 %)	13 (5 %)	255
2010	79 (31 %)	44 (18 %)	86 (34 %)	23 (9 %)	7 (3 %)	8 (3 %)	4 (2 %)	251
2011	73 (21 %)	65 (19 %)	116 (34 %)	42 (12 %)	39 (11 %)	6 (2 %)	4 (1 %)	345
2012	64 (20 %)	43 (13 %)	112 (34 %)	50 (15 %)	32 (10 %)	9 (3 %)	16 (5 %)	326
2013	65 (20 %)	42 (13 %)	110 (33 %)	43 (13 %)	43 (13 %)	13 (4 %)	12 (4 %)	328
2014	49 (17 %)	39 (13 %)	104 (36 %)	40 (14 %)	36 (12 %)	8 (3 %)	13 (5 %)	289
2015	50 (15 %)	50 (15 %)	119 (35 %)	57 (17 %)	34 (10 %)	13 (4 %)	14 (4 %)	337

*Penetr*. penetrating keratoplasty, *Lamel*. anterior lamellar keratoplasty, *Endoth*. endothelial keratoplasty, *Miscell*. miscellaneous

(Percentages of publications by year are written in parenthesis)

**Table 2:** Quantitative distribution and proportions of the publications according to the document types and study design

Year	OA & CR	Letter	MA	Review	Correction	Total
2006	133 (85 %)	12 (7.5 %)	6 (4 %)	4 (2.5 %)	2 (1 %)	157
2007	159 (79 %)	28 (14 %)	6 (3 %)	7 (3.5 %)	1 (0.5 %)	201
2008	183 (77 %)	39 (16.5 %)	11 (5 %)	3 (1 %)	1 (0.5 %)	237
2009	212 (83 %)	30 (12 %)	4 (1.5 %)	7 (3 %)	2 (0.5 %)	255
2010	212 (84.5 %)	34 (13.5)	0 (0 %)	5 (2 %)	0 (0 %)	251
2011	253 (73.5 %)	77 (22 %)	7 (2 %)	6 (2 %)	2 (0.5 %)	345
2012	246 (75.5 %)	52 (16 %)	10 (3 %)	15 (4.5 %)	3 (1 %)	326
2013	236 (72 %)	48 (14.5 %)	29 (9 %)	12 (3.5 %)	3 (1 %)	328
2014	216 (75 %)	31 (10.5 %)	23 (8 %)	19 (6.5 %)	0 (0 %)	289
2015	214 (63.5 %)	41 (12 %)	73 (21.5 %)	5 (1.5 %)	4 (1.5 %)	337

*OA* original articles, *CR* case reports, *MA* meeting abstracts

**Fig. 1:**
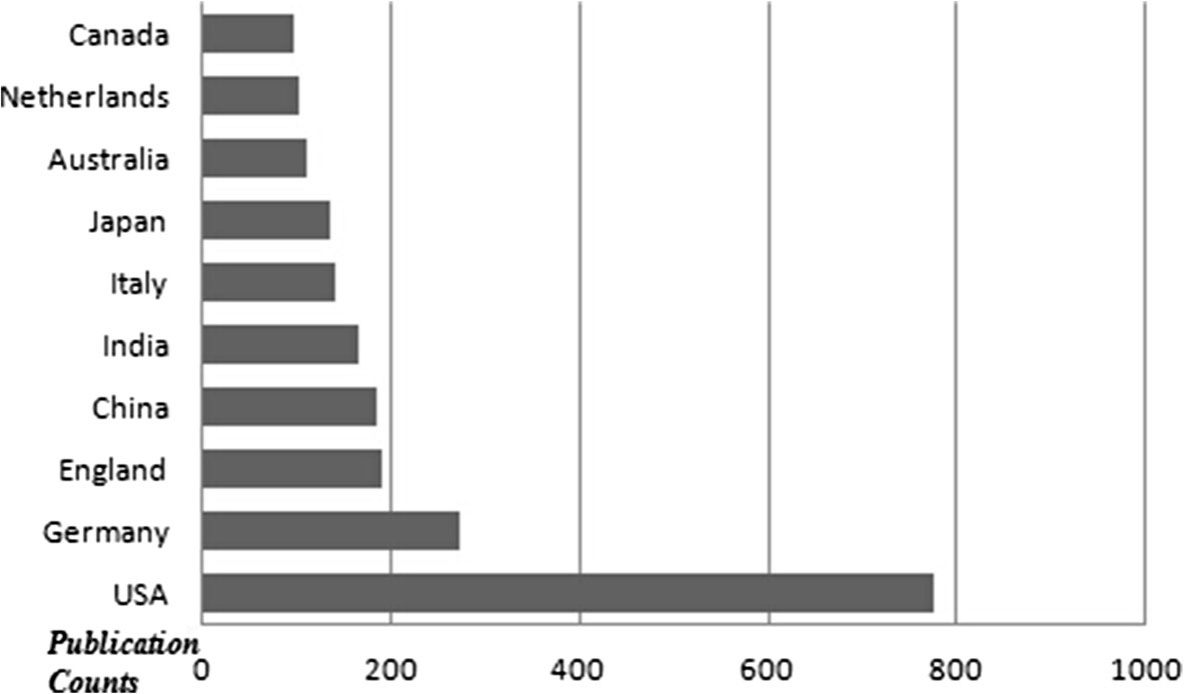
The top 10 countries which published the most of the keratoplasty documents are shown

**Fig. 2:**
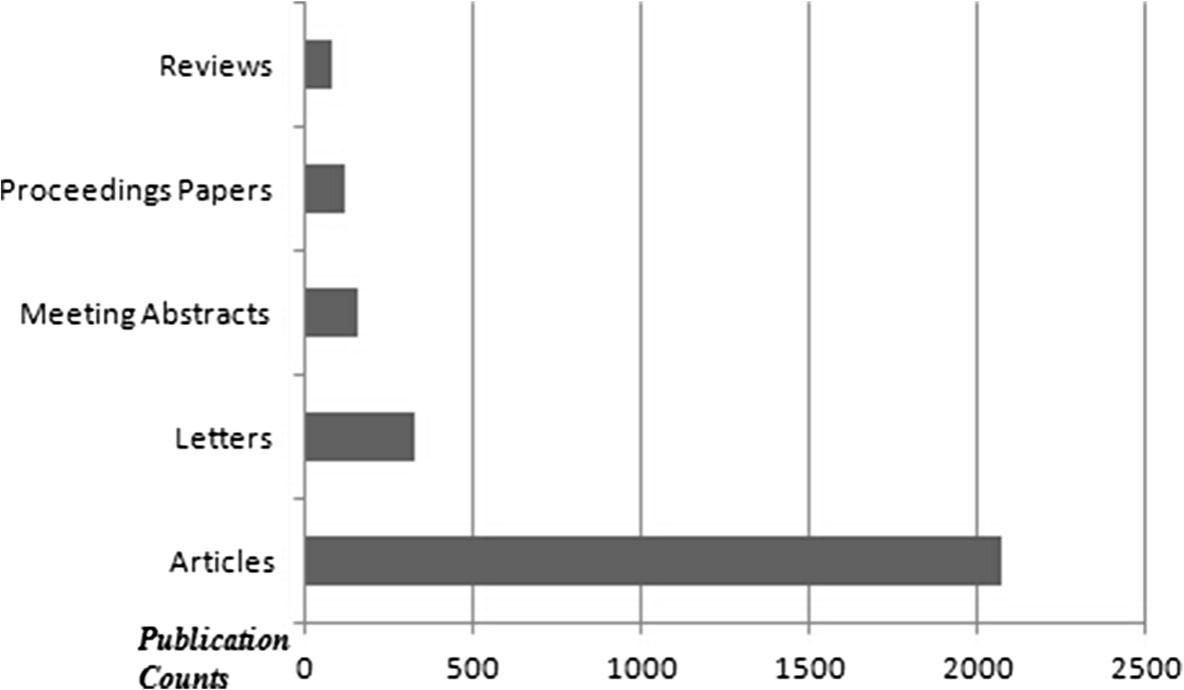
The top 5 document types are demonstrated (‘Articles’ represent original clinical, research or experimental studies and case reports)

**Fig. 3:**
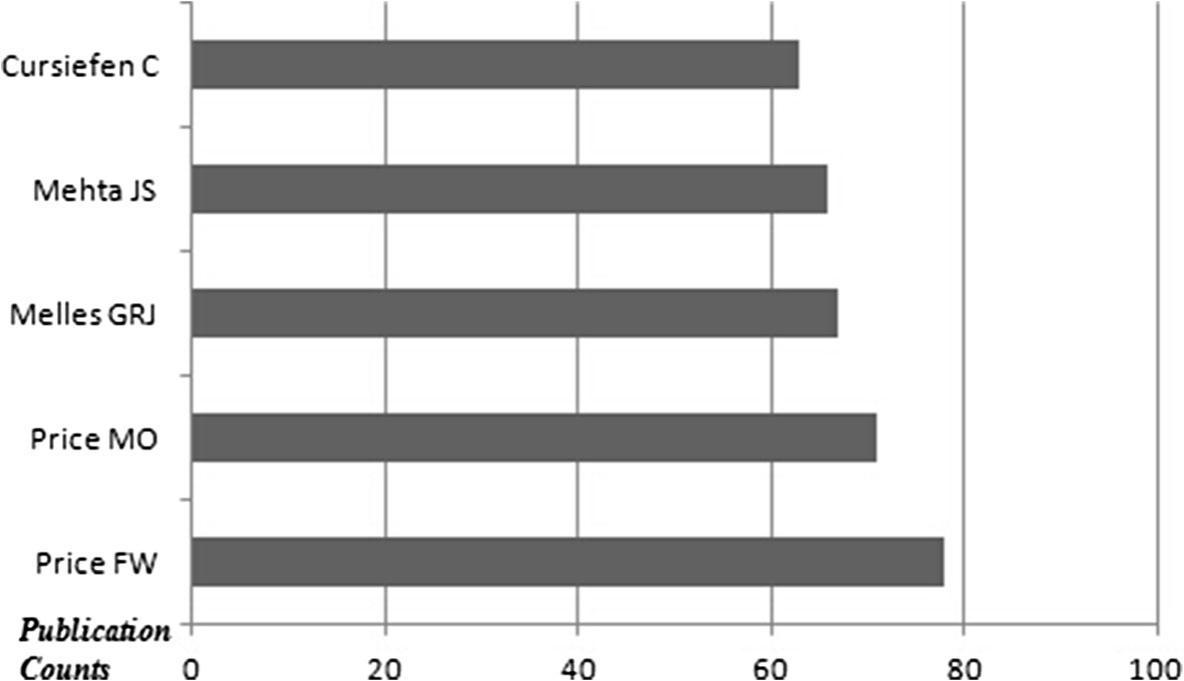
The top 5 authors’ names according to the published item count are shown

**Fig. 4:**
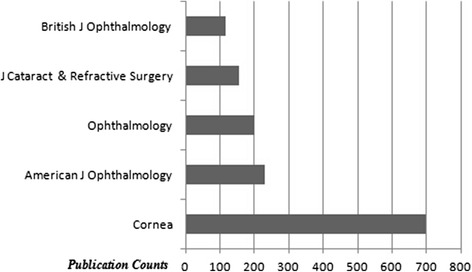
The top 5 journals which published the most of the keratoplasty documents are demonstrated

The top 10 countries which published the most of the documents are demonstrated in Fig. 1. The USA took the first place among the countries according to the amount of documents (approximately 28.4 %). The mostly used languages were English (2067 documents), German (109 documents) and French (27 documents). The top 5 document types are shown in Fig. 2. Original articles were the most published document types (approximately 75.5 %). The list of the authors who are on the top according to the published item count is shown in Fig. 3. Price FW and Price MO were the most productive authors about keratoplasty. The top 5 journals which published the most of the keratoplasty documents are demonstrated in Fig. 4. The ‘Cornea’ was on the top of list according to the amount of documents among journals (approximately 25.3 %).

The sum of the citations related to the keratoplasty documents published in the last decade was 25496. Average citations per item were 9.34. The year which had the most citation count was 2014 (a total of 4324 citations). The most cited article in the last decade about corneal transplantation was entitled ‘Descemet-stripping automated endothelial keratoplasty’ written by Gorovoy MS (approximately 400 citations).

## Discussion

Our results indicate that publication trends in corneal transplantation show tendency to posterior lamellar techniques. Although the lamellar keratoplasty techniques are more difficult to learn and perform, many corneal surgeons are keen on these procedures. Since undiscovered areas about lamellar techniques are many, it is likely to see growing number of publications in that topic.

From the year 2000 on, both keratoconus and Fuchs’ dystrophy have been among the most important indications of corneal transplantation [8, 9]. Introduction and success of anterior and posterior lamellar corneal transplantations had an influence on the trends in indications for penetrating keratoplasty [8]. Endothelial keratoplasty showed successful results for Fuchs’ dystrophy and anterior lamellar keratoplasty offered a good option for the treatment of keratoconus. Lamellar techniques (i.e., anterior lamellar keratoplasty, DSAEK, DMEK) became the treatment of choice in corneal diseases involving only certain layers of the cornea, because they offer less astigmatism or corneal graft rejection and better tissue rehabilitation [9–11]. Changing indications of corneal transplantation also changed the publication trends in this topic, since there are much more unknown issues related to the lamellar techniques when compared to penetrating keratoplasty. The growing number of publications related to the new keratoplasty techniques improves our understanding about the safety and efficacy of those procedures.

The publications related to keratoprosthesis and comparisons of different keratoplasty techniques have been increased in the last 5 years. The proportion of ‘animal studies’ stayed steady in the last decade. It is likely to see documents related to comparisons of different keratoplasty techniques, since several new methods have been described in the last decade. Keratoprosthesis surgery may be considered as an alternative to keratoplasty in some cases. Implantation of an artificial cornea (i.e. keratoprosthesis) is a good option for the eyes which has low success rates of corneal transplant surgery such as comorbid limbal stem cell deficiency due to chemical or thermal burns, viral keratitis, ocular cicatricial pemphigoid, trachoma and repeated transplantation [12–14].

The cornea was the first successful transplanted tissue more than 100 years ago [15]. Besides improvements in the surgical techniques, our understanding about the immunological aspects and pharmacotherapy of a corneal transplant has increased [16, 17]. With the introduction of new medical therapies, several papers related to immunology and pharmacotherapy of keratoplasty have been continuously published in the last decade. Our results also showed that the United States of America is the most productive country for keratoplasty publications. The Cornea is the leading journal about corneal transplantation. We also noticed that citations per keratoplasty documents may be considered satisfactory. The proportion of original articles and case reports slightly decreased by year in the last decade, and the proportion of letters stayed almost steady.

Our study has its limitations. First of all, it was not possible to read the full texts of all the published articles to give more details. Also the types of parameters evaluated in bibliometric studies are limited to the options given by the database programs. In further studies, more databases may be evaluated other than Thomson Reuters Web of Science. In a different point of view, one may say that increase in number of publications does not reflect increase in interest in corneal transplantation, because the amount of journals and so the published documents increase year by year. Since journal quality, acceptance rates, and acceptance methodology varies from journal to journal, it is likely that some manuscripts that not have been able to be published in 2006 were published in 2015. Lastly, we did not perform further work on subcategory formation for ‘animal studies’, since the amount of publications in this category is few.

## Conclusions

In summary, endothelial keratoplasty has been a hot topic in the recent years. There is a growing interest to this technique and the publications related to it outnumber the other corneal transplantation techniques. Also the total number of keratoplasty publications has been increased in the last years.

## Abbreviations

DMEK: Descemet membrane endothelial keratoplasty
DSAEK: Descemet stripping automated endothelial keratoplasty
SPSS: Statistical Package for the Social Sciences

## References

[1] Tan DT, Dart JK, Holland EJ, Kinoshita S (2012). Corneal transplantation. Lancet.

[2] Ple-Plakon PA, Shtein RM (2014). Trends in corneal transplantation: indications and techniques. Curr Opin Ophthalmol.

[3] Kim MH, Chung TY, Chung ES (2013). A retrospective contralateral study comparing deep anterior lamellar keratoplasty with penetrating keratoplasty. Cornea.

[4] Kitzmann AS, Wandling GR, Sutphin JE, Goins KM, Wagoner MD (2012). Comparison of outcomes of penetrating keratoplasty versus Descemet’s stripping automated endothelial keratoplasty for penetrating keratoplasty graft failure due to corneal edema. Int Ophthalmol.

[5] Holden G, Rosenberg G, Barker K (2005). Bibliometrics: a potential decision making aid in hiring, reappointment, tenure and promotion decisions. Soc Work Health Care.

[6] Luukkonen T (1990). Bibliometrics and evaluation of research performance. Ann Med.

[7] Cooper ID (2015). Bibliometrics basics. J Med Libr Assoc.

[8] Wang J, Hasenfus A, Schirra F, Bohle RM, Seitz B, Szentmáry N (2013). Changing indications for penetrating keratoplasty in Homburg/Saar from 2001 to 2010–histopathology of 1,200 corneal buttons. Graefes Arch Clin Exp Ophthalmol.

[9] Lang SJ, Bischoff M, Böhringer D, Seitz B, Reinhard T (2014). Analysis of the changes in keratoplasty indications and preferred techniques. PLoS One.

[10] Galvis V, Tello A, Gomez AJ, Rangel CM, Prada AM, Camacho PA (2013). Corneal transplantation at an ophthalmological referral center in Colombia: indications and techniques (2004–2011). Open Ophthalmol J.

[11] Glasser DB (2011). Changing trends in keratoplasty. Am J Ophthalmol.

[12] Jašinskas V, Rudalevičius P, Miliauskas A, Milčius D, Jurkūnas UV (2013). Keratoprosthesis surgery as an alternative to keratoplasty. Medicina (Kaunas).

[13] Magalhães FP, Sousa LB, Oliveira LA (2012). Boston type I keratoprosthesis: review. Arq Bras Oftalmol.

[14] Liu C, Hille K, Tan D, Hicks C, Herold J (2008). Keratoprosthesis surgery. Dev Ophthalmol.

[15] Kirkwood BJ (2006). A short history of corneal transplantation: commemorating 100 years. Insight.

[16] Pleyer U, Schlickeiser S (2009). The taming of the shrew? The immunology of corneal transplantation. Acta Ophthalmol.

[17] Niederkorn JY (2015). Immunology of corneal allografts: insights from animal models. J Clin Exp Ophthalmol.

